# Exploring Treatment Completion and Participant Feedback in an Adapted Intervention among Incarcerated Men with Mental Illness

**DOI:** 10.1007/s10597-025-01539-9

**Published:** 2025-10-17

**Authors:** Melissa L. Villodas, Jonathan Phillips, Ehren Dohler, Anna Parisi, Faith Scanlon, Chloe Pilkerton, Amy Blank Wilson

**Affiliations:** 1 Department of Social Work, George Mason University, 4400 University Drive, MSN IF8, 22030 Fairfax, VA USA; 2 Department of Social Work, University of Minnesota Duluth, Duluth, MN USA; 3 School of Social Work, University of North Carolina at Chapel Hill, Chapel Hill, NC USA; 4 Department of Psychiatry, Massachusetts General Hospital, Harvard Medical School, Boston, MA USA

**Keywords:** Intervention research, Mental illness, Criminal legal system, Treatment retention, Criminogenic factors

## Abstract

Treatment completion in interventions within correctional settings is challenging both generally and for people with mental illness (MI). Few studies have explored treatment completion alongside feedback from this population. We examined the rates of treatment completion alongside participant feedback to explore how participant feedback can improve treatment completion. Mixed-methods were used to examine treatment completion rates and clinical characteristics of participants (*n* = 24), and to explore participant feedback. We used thematic, qualitative analysis of participant feedback (*n* = 17) obtained from interviews, alongside univariate statistics to describe the sample of treatment completers and non-completers. Three-quarters of participants completed the treatment. Non-completers had a higher proportion of one or more infractions than completers. Four themes from participant feedback emerged in the qualitative analysis: *Practice*,* Applicable Takeaways*,* Intervention Pace*,* and Group Dynamics*. Participants’ feedback highlights important considerations for responsive practices to improve treatment completion among incarcerated men with MI.

Challenges in treatment retention abound among criminal legal system interventions for people with mental illness (MI). While several studies have explored the predictors of treatment dropout (Bennemann et al., 2022; Cullen et al., 2011 ), few have incorporated participant feedback to improve our understanding of treatment participation from the perspective of treatment recipients. This research is particularly important for individuals with MI (i.e., Schizophrenia spectrum disorders and major affective disorders) who are overrepresented in the criminal legal system. Indeed, up to 30% of individuals incarcerated in jails have an MI (Crilly et al., 2009; Prins, 2014; Steadman et al., 2009; Teplin et al., 1996; Wilson et al., 2018). Research demonstrating high levels of risk factors for criminal legal system involvement, or *criminogenic neeeds* (e.g., antisocial behavior, personality, cognition, and associates; Bonta & Andrews, 2023), among people with MI has recognized the need for services for this population to include interventions that directly target the risk factors most closely associated with recidivism (Morgan et al., 2010; Skeem et al., 2011, 2014; Wilson et al., 2014; Wolff et al., 2011, 2013). Over the years, researchers have adapted and developed interventions that address these criminogenic risk factors (Epperson et al., 2014; Morgan et al., 2018; Batastini et al., 2021; Wilson et al., 2018). For example, interventions like Changing Lives, Changing Outcomes (Morgan et al., 2018) and Stepping Up Stepping, Stepping Out (Batastini et al., 2021) integrate psychotherapeutic and psychoeducational strategies that focus on both criminogenic and psychiatric risk for incarcerated people with MI and have been found to be effective in reducing both mental health symptomatology and criminal risk (Gaspar et al., 2019; Batastini et al., 2021).

These interventions are grounded in the Risk-Needs-Responsivity (RNR) model, which is guided by core principles that enhance and strengthen the design and implementation of effective interventions targeting criminogenic risk (Bonta & Andrews, 2023). The three core principles are: the Risk Principle (i.e., match the level of service to one’s risk to re-offend), the Need Principle (i.e., assess criminogenic needs and target them in treatment), and the Responsivity Principle (i.e., maximize the individual’s ability to learn by providing cognitive behavioral treatment and tailoring the intervention to the learning style, motivation, abilities and strengths of the offender) (Bonta & Andrews, 2023; Bonta & Andrews, 2007).

## Responsive Treatment Approaches and Treatment Completion

Mental health services research has long established that individuals with MI have historically experienced low levels of treatment participation (see Dixon et al., 2016), which is often quantified in intervention studies as rates of treatment completion (Cullen et al., 2011, 2012; Rees-Jones et al., 2012). Evidence suggests that factors such as psychiatric symptomatology, impaired cognition, low interpersonal skills and self-efficacy, and heightened levels of distress and disorganization may contribute to low levels of treatment participation among individuals with MI generally (Dixon et al., 2016; Drapalski et al., 2008; Velligan et al., 2017). These factors may also drive especially low completion rates (cited at 50%; Cullen et al., 2011, 2012) found among interventions delivered in correctional settings with individuals with MI compared to other non-mental health prison programming like vocational, GED, and Therapeutic Communities (completion rates ranging from 72 to 83%; see Nur, 2025).

Low completion is concerning, as treatment completion is associated with better treatment outcomes among clients receiving cognitive-behavioral therapy generally (Cahill et al., 2003; Lamproproulos, 2010) and clients involved in the criminal legal system (see Kroner et al., 2014; Kroner & Takahashi, 2012), as higher treatment dosage allows clients more time to learn and benefit from programming. Research suggests that criminogenic-focused interventions that employ responsive treatment approaches – interventions that match the learning styles and abilities of offenders and moderates an individual’s response to treatment by focusing on challenges they may navigate, such as reasoning skills (Bonta & Andrews, 2023; McCormick et al., 2015) – are successful in improving treatment completion among this population. However, even with RNR principles incorporated, some studies continue to see variability in completion scores. For example, Reasoning and Rehabilitation (R&Ross et al., 1988) has been adapted to be delivered to individuals with MI across several studies, with reported completion rates ranging from 50% to 84% across nine studies (see review by De Ribera et al., 2024). Maximizing the ability of individuals with MI to participate in criminogenic-focused interventions may be a key ingredient to improving criminal legal system outcomes for this population (Cullen et al., 2012). A recent small-scale pilot study by Wilson and colleagues (2023; *N* = 47) reported promising findings for an adapted version of Thinking for a Change (T4C; Bush et al., 2011), an established criminogenic-focused intervention, for incarcerated people with MI. This adaptation was implemented via a novel Targeted Service Delivery Approach (TSDA), a structured therapeutic framework encompassing five strategies designed to modify the delivery of interventions like T4C to address the specific responsivity needs of this population and maximize participation to enhance their treatment engagement and support completion (Wilson et al., 2018).

## The Current Study

While there is broad agreement that treatment participation is necessary for criminogenic-focused interventions to be effective (Hollin et al., 2008; McMurran & Theodosi, 2007; Rees-Jones et al., 2012), little is known about the clinical and criminal legal characteristics of participants alongside feedback from intervention participants that may support treatment completion (O’Brien & Daffern, 2017; Ward et al., 2007) and how to tailor these interventions to be more responsive to the needs of incarcerated individuals from the perspective of participants. Therefore, the current study employs a mixed methods approach using data from the treatment arm of a small-scale RTC of a criminogenic-focused intervention for incarcerated men with MI to address the following questions: (1) What was the treatment completion rate for a criminogenic-focused intervention for people with MI in prison? (2) What are the clinical characteristics of study participants who completed the study intervention and of those who did not? (3) How does participant feedback on the intervention help inform our understanding of treatment completion? This study contributes to the limited body of research examining treatment completion and participant feedback among incarcerated individuals with MI, a population with significant and often unmet treatment needs in prisons. While much of the existing literature focuses on barriers to treatment completion or reasons for dropout, this study shifts attention to solutions by combining descriptive data on rates of completion with qualitative feedback.

## Method

### Study Intervention

Data for the current study were drawn from the treatment arm of a small-scale pilot study (hereafter referred to as T4C-TSDA) which investigated a criminogenic-focused intervention newly adapted for people with MI incarcerated in prison. A description of the research methods used in the clinical trial is available on *ClinicalTrials.gov* (Identifier: NCT03713398). All research associated with this study was approved by the Institutional Review Board at the University of North Carolina at Chapel Hill.

The adapted intervention, referred to as T4C-TSDA, comprises two components. The first component is T4C (version 3.0; Bush et al., 2011), an established, manualized CBT program lasting approximately 14 weeks and delivered to 8–12 participants in a closed-group format, held at least twice a week for 1 h per meeting. The second component is the TSDA (Wilson et al., 2018), which functions as the adaptation mechanism for tailoring the delivery of T4C to address the distinct learning and treatment needs of individuals with MI. In this study, T4C provided the intervention *content*, and the TSDA provided a therapeutic framework for modifying the *delivery* of this content to maximize the ability of this population to fully engage in and benefit from the underlying intervention (Wilson et al., 2018).

The TSDA’s therapeutic framework is built upon five therapeutic delivery strategies applied throughout each intervention session: repetition and frequent summarizing (maximizing knowledge acquisition by regularly restating key concepts and information); amplification techniques (breaking down abstract information into concrete examples); active coaching (the provision of individualized coaching to support knowledge acquisition and skill development); low-demand practice (providing opportunities to practice skills in a manner that minimizes behavioral expectations and positively reinforces approximations of the intended behavioral change); and maximizing participation (using techniques to increase motivation to attend and engage in the group while enhancing participants’ ability to carry out newly learned skills) (Wilson et al., 2018).

### Sample

The present study focuses on the subset of participants randomly assigned to the experimental T4C-TSDA condition within the broader parent study, which took place in a medium-security men’s prison located in the southeastern United States. The intervention was delivered across two cycles in this prison by two research staff members who had a master’s degree in social work (MSW) or an equivalent degree between January 2019 and December 2020. Recruitment for each cycle began approximately one month prior to the intervention start date and continued until the target sample size was achieved.

To be included in the study, participants, all of whom were men, had to: (1) be aged 18 years or older; (2) have a diagnosis of bipolar disorder, major depressive disorder, schizophrenia spectrum disorder, or other psychotic disorder; (3) have moderate or higher criminogenic risk levels as determined by the Level of Service Inventory (LS/CMI; Andrews et al., 2004), and (4) have at least one year or more remaining in their prison sentence at the time of the screening interview. Study exclusion criteria included: (1) having an intellectual or developmental disability; (2) assault precautions or other restrictions that would preclude the person from being in group gathering spaces within the prison where the intervention took place; and (3) participation in T4C within the last 6 months.

At the start of each recruitment cycle, correctional staff provided study personnel with a list of potentially eligible individuals. Study staff completed all recruitment activities, which included meeting with potential participants in private settings within the prison facility, providing potential participants with study flyers, and answering questions. Study staff completed informed consent with potential participants who expressed interest. Study staff then completed screening interviews with potential participants who provided informed consent to determine study eligibility. Study staff emphasized the voluntary nature of the study throughout all steps in the recruitment process. Eligible participants who agreed to participate and met the study eligibility criteria were subsequently randomized to either the experimental T4C-TSDA condition (*n* = 24) or standard prison treatment and programming (*n* = 23). For a detailed description of this clinical trial’s recruitment and enrollment procedures, see Phillips et al. (2023). The present analysis includes only those randomized to receive T4C-TSDA.

### Data Collection

Data for the current study were drawn from three sources. Quantitative data from the study’s screening interview were used to examine the demographic and clinical characteristics of study participants. Screening interviews for the clinical trial were conducted by members of the study team (i.e., the first, second, fourth, and last author). Administrative records were used to evaluate criminal charges and track criminal legal outcomes during the parent study’s intervention period (i.e., from the study baseline through three-month follow-up). Finally, qualitative data from the 3 month follow up interviews were collected by two research team members who did not facilitate the intervention (i.e., the first and second authors). Individual face-to-face interviews lasting 60–90 min were conducted during the study’s three-month follow-up interview, which took place after the study intervention was completed. During this interview, participants were asked three open-ended questions: (1) What did you find useful about the T4C program? (2) What did you find challenging about the T4C program? and (3) What suggestions do you have for how the T4C program could be improved? Responses to these questions were written down verbatim by the data collector at the time of the interview. Responses were subsequently transcribed, entered into Qualtrics, and transferred to Microsoft Excel for analysis.

### Measures

Demographic, clinical, and criminal legal system data were collected during the screening interview, as well as from prison administrative records. Participants’ age and number of years incarcerated were determined via self-report. Mental health diagnosis was determined through the administration of the Mini-International Neuropsychiatric Interview (MINI; Sheehan et al., 1998). The MINI has demonstrated good reliability (κ = 0.75 or higher across diagnoses; Sheehan et al., 1998) and has been used in correctional settings (Fovet et al., 2025; Black et al., 2004). Mental health symptomatology was measured using the 18-item Brief Psychiatric Rating Scale (BPRS; Andersen et al., 1989). The BPRS has demonstrated acceptable reliability (α = 0.75) with correctional populations (Cloyes et al., 2006) and demonstrated acceptable reliability within the present study sample (α = 0.61; Bujang et al., 2018). Following anchor points for the BPRS as suggested by Lovell and Jemelka (1998), the following classification of total scores is based on level of impairment: 0 to 13 = mild impairment; 14 to 23 = moderate impairment; 24 to 36 = marked impairment; 37 + = severe impairment.

Having a “high” Criminogenic Risk Level was determined by a score of 20 or greater on the Level of Service/Case Management Inventory (LS/CMI; Andrews et al., 2004). The LS/CMI has demonstrated good reliability in a correctional sample of men (α = 0.89). Prison administrative records were utilized to ascertain (a) the occurrence of disciplinary behavioral infractions – measured dichotomously to indicate receipt of at least one disciplinary behavioral infraction – among participants during the pilot study intervention period as a criminal legal outcome and (b) whether participants had a primary charge of a violent offense leading to their incarceration.

Each of the measures mentioned above was analyzed separately based on treatment completion status. Following established conventions, treatment completion was defined as attending at least 80% of intervention sessions (Cullen et al., 2012; C-Y Yip, 2013; Rees-Jones et al., 2012; Young et al., 2016).

### Analysis Plan

#### Univariate Analysis

Univariate descriptive statistics were conducted on data from the study’s screening interview to describe the demographic and clinical characteristics of participants who did and did not complete treatment. Measures of central tendency were used to describe continuous variables (i.e., age, time incarcerated, and mental health symptomatology), while percentages were used to describe categorical variables (i.e., mental health diagnosis, receipt of a disciplinary behavioral infractions [collected from the study baseline through three-month follow-up], having a high criminogenic risk level, and being incarcerated for a violent offense).

#### Qualitative Analysis

The research team used a thematic and inductive two-step coding strategy to analyze the feedback that participants provided about the study intervention during the three-month follow-up interview (Braun & Clark,^2012^). In the first step of the analysis, two members of the research team (i.e., the first and third authors) used line-by-line open coding techniques (Emerson et al., 2011) to develop a list of descriptive codes. The coders met three times during this step to compare line-by-line coding notes and to apply the constant comparative method to refine their understanding and interpretation of the data, leading to clarified definitions for the emerging dimensions (Richards & Hemphill, 2018).

In the second step, these dimensions were grouped into larger analytic themes that were related to different structural components of the study intervention and therapeutic. The coders three times during this step, which was when they reached consensus on the organization of relevant dimensions into themes (Miles et al., 2020). A deductive element was integrated into the analysis during the second step, where coders linked the TSDA strategies to the relevant dimensions. To strengthen analytic rigor, member checking was conducted with the facilitators of the intervention to confirm the credibility of analytic interpretations (Hill et al., 2005; Padgett, 2017).

## Findings

### Univariate Analyses

Univariate analyses were used to describe the sample’s demographic, clinical, and criminal legal characteristics. 75% of the participants attended at least 80% of the intervention sessions and were therefore considered “treatment completers.” The mean age of the study sample (*N* = 24) was 40.8 years (*SD* = 10.0). The mean number of years participants were incarcerated was 12.2 (*SD* = 8.2). At the time of the screening interview, the sample’s mean BPRS score (i.e., psychiatric symptomology) was 32.3 (*SD* = 8.0), indicating marked impairment. On average, treatment completers were younger than treatment non-completers (M = 38.8, SD = 8.6 and M = 46.8, SD = 12.3, respectively). We also found that treatment completers were on average incarcerated for fewer years than treatment non-completers (M = 11.5, SD = 7.9 and M = 14.2, SD = 9.4, respectively). Average BPRS scores between treatment completers and treatment non-completers were about the same between the two groups (M = 32.1, SD = 8.1 and M = 32.8, SD = 8.4, respectively).

Roughly one-third of study participants had a diagnosis of schizophrenia spectrum disorder or other psychotic disorder – nearly two-thirds of whom were treatment completers. Per the LS/CMI, all treatment non-completers had a high (i.e., a score of 20 or greater) criminogenic risk score (M = 27.3, SD = 3.4), while roughly 75% of treatment completers had a high criminogenic risk score (M = 24.8, SD = 6.3). Approximately 50% of both treatment completers and non-completers were incarcerated for violent offenses. Finally, approximately half of treatment non-completers received a disciplinary behavioral infraction during the study intervention period, while roughly 10% of treatment completers received a disciplinary behavioral infraction.

### Qualitative Analysis of Participant Feedback

A total of 17 out of 24 participants responded to the qualitative questions. Almost 90% of these respondents were treatment completers, whereas just under 50% of the individuals who did not respond to qualitative questions were treatment completers. The qualitative analysis identified four overarching themes in participant feedback: *Practice*, *Applicable Takeaways*, *Intervention Pace*, and *Group Structure and Dynamics*. Each theme is described in detail below in Table 1, with its associated dimensions.

**Table 1:** Participant feedback on intervention

Themes	Dimensions
Practice	Role Playing (AC)(AT) Repetition (RS) Intervention Materials (AT)
Applicable Takeaways	Empathy Problem Solving Goal Setting
Intervention Structure	Pacing (MP) Group Size
Group Dynamics	Group Cohesion Facilitator Presence

Note. TSDA strategies are denoted by abbreviations. Repetition and summarizing (RS), amplification techniques (AT), active coaching (AC), maximize participation (MP)

#### Practice

Participants’ feedback was categorized as *Practice* theme when participants discussed how the intervention provided or could have provided opportunities for them to improve their mastery of the intervention content. This theme included three dimensions reflecting aspects of the study intervention that facilitated participants’ ability to practice: role-playing, repetition, and intervention materials. Participants identified role-playing as an important aspect of practice in the study intervention. For example, Participant 15 expressed that they “liked the role-playing,” and Participant 10 indicated that they wanted “more role-playing for participants.” Building on the theme of Practice, Participant 12 stated that they were “concerned that the group [study intervention] is over and want[ed] to practice more.” Along those same lines, other participants felt that regular follow-up and the development of advanced classes would help facilitate more opportunities to practice what they had learned through their participation in the intervention. For example, Participant 10 stated they “would like to have an advanced class,” and Participant 8 discussed his desire for advanced classes to participate in after the intervention ended, recommending a “follow-up to use the skills.”

Participants also provided feedback about intervention materials like didactic handouts and worksheets that aid in practice both within and outside of the intervention space. For example, some participants indicated that they would like materials they could use for practice after the group was over. Participant 7 stated a “resource booklet to keep going forward or booklet to work through during the program” would have supported their ability to practice what they were learning in the study intervention. Similarly, Participant 5 stated that there were “a lot of handouts to keep up with. [I] recommend making a book people could use throughout the course.”

#### Applicable Takeaways

Participant feedback was categorized under the theme of *Applicable Takeaways* when participants noted something that they learned in the study intervention they felt was useful, relevant, and easily translatable to other situations in their lives. The key dimensions of feedback that emerged from this theme included empathy, problem-solving, and goal setting. With respect to empathy, Participant 9 stated they liked that the intervention “taught him empathy for different people” and “to control conflict in situations.” Participant 7 highlighted the value of the intervention in teaching him problem-solving skills, stating how it “helped provide option[s] to deal with situations.” Participant 18 stated that they found “sticking to objective facts was helpful during the classes” when learning problem-solving skills, describing how he appreciated the intervention’s “practical breakdown of material.” Participant 1 emphasized the importance of goal setting, noting that he found it useful that he “could set goals when in a conflict situation.”

#### Intervention Structure

Participant feedback was categorized under the theme of *Intervention Structure* when participants noted structural aspects of the study intervention that they thought worked well or that could be improved. Two dimensions emerged under this theme: the pacing of the intervention content and group size. Overall, participants’ feedback about the pacing of the intervention content, which includes the time, duration, and frequency of the intervention, was positive. For example, Participant 8 stated that holding sessions “twice a week was good.” However, there was diversity in feedback related to the pacing of intervention content. Some participants shared that they would have liked more engagement with the intervention. For example, Participant 2 stated that while they believed the “time of day was good,” they “would have liked [the intervention sessions] to be longer,” and Participant 3 thought the study team “could run it [the intervention] more times per week.” Conversely, Participant 10 “found the lessons to be a bit long,” while Participant 12 believed “the pace was pretty fast,” highlighting that there was no one-size-fits-all approach to pacing of intervention content. For group size, Participants 2 and 8 both agreed that there were a “good number of people” in the group, with Participant 4 specifically stating that they “liked [that] it was a small group.”

#### Group Structure and Dynamics

Finally, participant feedback was categorized as the *Group Structure and Dynamics* theme when participants noted dynamics in the group that they thought facilitated support and a space for authentic connection. Dimensions related to group structure and dynamics included group cohesion and facilitator presence. For group cohesion, Participant 19 stated “the group members have rapport with each other. Everyone is dealing with some of the same problems.” Similarly, participant 20 stated “It’s realistic, we don’t try to fake it.” Participant 9 highlighted the benefits of going through the intervention as a group, stating that they “like working as a team with others to solve problems.” Participants also discussed feeling supported and connected with other members of the group, which encouraged them to openly express their desires. Participant 1 stated “People want space to open up. People felt safe.” While most participants spoke favorably about the group dynamics, one participant (Participant 12) noted the need for facilitation strategies to encourage equal opportunities for engagement among all group members, describing how “I didn’t get a chance to share. People should raise their hands.” Other participants described how the communication styles and demeanors of facilitators positively impacted their experience within the group. For example, Participant 15 stated they “like how facilitators interact with people.” Similarly, Participant 19 noted that “facilitators have great personalities,” and Participant 20 noted that “staff is friendly.”

## Discussion

In this study, consisting of 24 incarcerated men with MI, we utilized mixed methods to examine treatment completion rates, characteristics of treatment completers and treatment non-completers, and feedback from participants (*N* = 17) about their experiences participating in T4C-TSDA, an adaptation of T4C which explicitly focused on maximizing participants’ ability to engage in and benefit from the intervention content. Participants’ feedback from qualitative interviews offers insights that can be used in criminogenic-focused interventions to support treatment completion, as well as other ways existing criminogenic-focused interventions can be more responsive to the needs of people with MI.

Given the alignment of the quantitative and qualitative findings with the TSDA (a novel and important component of this study intervention), the findings are contextualized within this framework. With previous research highlighting the elevated rates of treatment drop-out among people with MI in the criminal legal system (e.g., Ashford et al., 2008; Cullen et al., 2011, 2012), the high rates of treatment completion in this study (75%) are promising. Within our study sample, mental health symptomology scores appeared to be about the same among both treatment completers and treatment non-completers. We found that most individuals with schizophrenia spectrum disorder or other psychotic disorder completed treatment, suggesting the TSDA adaptations to T4C may have positively supported attendance. It is important to note that our administrative data did not include treatment adherence or medication management outside of T4C-TSDA. Future studies may benefit from examining these trends alongside complementary services that may support treatment completion among incarcerated men with schizophrenia spectrum disorder or other psychotic disorder.

In contrast to mental health diagnosis trends, we found that the participants who did not complete treatment all had high criminogenic risk levels (i.e., a score of 20 or greater on the LS/CMI) and had a higher proportion of one or more disciplinary behavioral infractions than the treatment completers during the intervention period. These findings may signal that the people who may have benefited from the intervention the most may have been the hardest to retain in this intervention. In other words, individuals who are most at risk of breaking prison rules may be least likely to be able to fully engage in the full course of the intervention. This is consistent with Olver et al. (2011), who found that treatment recipients with high criminogenic risk and needs were less likely to complete treatment.

In light of the criminogenic risk and responsivity challenges that may be associated with treatment non-completers (e.g., low motivation, disruptive behavior, limited engagement), it is important to account for additional considerations in program implementation. While the facilitators of T4C-TSDA were MSWs trained to work with incarcerated men with MI, staff delivering such interventions should be prepared to anticipate challenging behaviors, appropriately monitor countertransference that may emerge, and leverage existing guidelines and resources for working with incarcerated people with high criminogenic risk (Olver et al., 2011; Wong & Hare, 2005). Beyond facilitator considerations, which were positively regarded in this study, it is also important to consider how the structure and delivery of criminogenic interventions in prisons may impact participation among individuals with MI who have high criminogenic risk levels. While the TSDA’s delivery strategies are designed to reduce engagement barriers among people with MI, those with high criminogenic risk may have additional responsivity needs that require support. It is also possible that this subgroup may benefit from modified content or alternative intervention structures to better support their participation. For instance, evidence suggests that incorporating motivational interviewing into criminogenic interventions can improve session attendance and reduce dropout compared to interventions lacking this component (e Silva et al., 2025). Future research should work to disentangle the effects of facilitator characteristics, intervention content, and delivery strategies to identify the factors that promote treatment completion among individuals with MI and elevated criminogenic risk.

Similar to our findings that high criminogenic risk scores were present among the treatment non-completion group, we found that disciplinary behavioral infractions were high among treatment non-completers. While structures that enforce disciplinary consequences for infractions may be necessary to ensure safety and accountability, these results suggest that a balance may need to be found between the need for security and the need for treatment in correctional facilities. Prioritizing, or simply supporting, sustained treatment participation may help address the underlying issues that contribute to safety concerns within correctional settings. Taken together, findings related to high criminogenic risk and the higher proportion of disciplinary behavioral infractions among treatment non-completers highlight the need for future research to examine their relationship and inform strategies to minimize treatment dropout due to disciplinary behavioral infractions.

While the participants were not asked specifically about the TSDA strategies as part of their feedback, several of the qualitative themes that emerged from this feedback closely align with the strategies of maximizing participation, amplification, and low-demand practice—strategies that are explicitly designed to address responsivity needs among individuals with MI. One opportunity presented through participant feedback that may address high criminogenic risk scores and the higher proportion of disciplinary behavioral infractions is through practice opportunities. Research indicates the importance of encouraging practice during group and outside of group generally (i.e., “homework”; see Kazantzis & Miller, 2022) and when implementing criminogenic-focused interventions (see Morgan et al., 2002). The TSDA’s low-demand practice strategy encourages active participation by providing chances for skill practice without strict expectations for what constitutes a “successful” effort, thus engaging therapeutic techniques that reinforce all efforts toward behavioral change. Given prior research showing challenges with homework adherence in CBT interventions, even among individuals without MI (Kazantzis et al., 2005), participant feedback requesting more practice opportunities and ongoing support, as well as post-group follow-up sessions, is encouraging and indicates the potential effectiveness of this approach in promoting sustained engagement. These findings highlight the potential of TSDA strategies to reduce responsivity-related barriers and promote engagement in criminogenic interventions for incarcerated individuals with MI.

Regarding the study intervention’s structure and delivery, participants expressed satisfaction with the group size, frequency, and timing. These findings align with the TSDA strategy of maximizing participation, which aims to enhance motivation and self-efficacy among individuals to actively engage in group activities and apply newly learned skills (Wilson et al., 2018). Core techniques of this strategy involve maintaining small group sizes and ongoing collaboration with participants to identify and address engagement obstacles accordingly. For instance, adjusting session lengths to match participants’ cognitive capacities is one way to overcome these barriers. Although feedback on the intervention’s pace was generally positive, there was variability among participants. These findings are consistent with previous TSDA research, emphasizing the importance of tailoring intervention to the needs of the group and gathering feedback on session pacing (Wilson et al., 2018). While it will not always be possible to identify a single intervention pacing that meets the needs of all participants, flexible and responsive delivery of the treatment through four of the five TSDA strategies (e.g., repetition and summarizing, low demand practice, amplification techniques, and active coaching) may be leveraged to support participants in this area.

In addition to intervention structure and delivery, the TSDA strategy of maximizing participation also involves techniques aimed at fostering positive relationships and group cohesion. Our findings suggest that participants valued the sense of cohesion and relationships within the group. Group activities, like opening and closing exercises in each session to foster a sense of identity and positive connections among participants, may have contributed to participants’ positive perceptions of the intervention and subsequent attendance. Furthermore, despite the intervention’s focus on challenging topics like criminogenic risk factors, participants found the group sessions engaging, as evidenced by the participants’ feedback and the study’s completion rate. Relatedly, participants’ positive perceptions of the intervention facilitators may also be indicative of a therapeutic alliance between themselves and the intervention facilitators, which has been shown to positively impact treatment improvement (Scanlon et al., 2022) and completion (see Blasko et al., 2018) in correctional settings, reduce recidivism (Sturm et al., 2021), and maximize participation.

Finally, another TSDA strategy that emerged through our qualitative analysis was amplification, which aims to enhance knowledge and skill acquisition by using multiple therapeutic strategies that focus on making abstract information into concrete examples. Amplification also aligns with the responsivity principle of maximizing the participants’ ability to learn by tailoring the intervention to the learning style, motivation, abilities, and strengths of the participant (Bonta & Andrews, 2007). To accomplish this, facilitators engaged in several strategies, including the use of handouts and flip charts (i.e., large Post-it notes on easels) to visually illustrate and clarify concepts. In this study, the facilitator’s visual aid options were regulated by facility-specific guidelines on what could and could not be brought into the prisons. Therefore, when planning to employ amplification techniques in correctional settings, it is crucial to both elicit participant feedback and collaborate with prison staff around navigating facility regulations (Wilson et al., 2018).

### Limitations

This study’s findings should be considered in light of limitations. First, this study used data from three open-ended questions embedded within a larger interview. As such, these were not in-depth interviews, nor were interviewers able to ask follow-up or probing questions to elicit more detailed information from participants regarding their responses to the questions. Despite this limitation, this study fills a gap in research by exploring feedback from incarcerated people with MI who are the end users of these criminogenic interventions. As our study is preliminary, future research should incorporate a more robust exploration of the opinions of incarcerated people with MI to enhance service delivery.

Second, the treatment groups were conducted among men, yet research has consistently found that gender represents an important responsivity issue and that women have unique needs that must be considered in the design and delivery of criminogenic interventions (Gobeil et al., 2016). As such, future research should investigate whether the engagement strategies highlighted in the present analysis may apply to incarcerated women with MI.

Third, regarding mental health, our inclusion criteria were limited to incarcerated men diagnosed with bipolar disorder, major depressive disorder, schizophrenia spectrum disorders, or other psychotic disorders. Individuals with comorbid personality disorders were excluded from the parent study, which limits the variability that may have emerged in this study related to disciplinary behavioral infractions and criminogenic risk.

Finally, the current study’s sample size was small as it comprised a single arm of a small-scale RCT. Given the small sample size, this study was not adequately powered to conduct bivariate tests of difference. Future research drawing from larger sample sizes should be conducted to examine similarities or differences that may emerge in the predictors of treatment completion in criminogenic-focused interventions among people with MI incarcerated in jail or prison.

### Conclusions

The results of this research demonstrate that criminogenic-focused interventions can achieve high completion rates with people with MI. While prior studies have explored predictors of treatment dropout, this study is among the first to integrate participant feedback with treatment completion data and clinical characteristics to inform more responsive practices that support treatment retention. Collectively, these findings offer practical insights that criminogenic-focused programs can consider to increase participants’ attendance and engagement in treatment by maximizing opportunities for active participation, fostering group cohesion, and implementing both program- and systemic-level adjustments that meet the treatment needs of incarcerated men with MI and support treatment completion.
